# The Effect of Conventional and Mini-Invasive Cardiopulmonary Bypass on Neutrophil Activation in Patients Undergoing Coronary Artery Bypass Grafting

**DOI:** 10.1155/2012/152895

**Published:** 2012-02-19

**Authors:** Martina Kolackova, Jan Krejsek, Vladimir Svitek, Pavel Kunes, Jiri Mandak, Zdenka Holubcova, Vladimir Lonsky

**Affiliations:** ^1^Department of Clinical Immunology, Faculty of Medicine, Charles University, 50005 Hradec Kralove, Czech Republic; ^2^Department of Cardiac Surgery, University Hospital, 50005 Hradec Kralove, Czech Republic

## Abstract

Interleukin-10 (IL-10) is considered to be a cytokine with potent anti-inflammatory properties, which have been previously linked to increased incidence of sepsis. The level of IL-10 is elevated by cardiac surgery when cardiopulmonary bypass (CPB) and methylprednisolone are used. In our study, we compare the level of IL-10, IL-10 Receptor (IL-10R), and percentage of neutrophils between two groups of cardiac surgical patients undergoing Coronary Artery Bypass Grafting, both of which were not given methylprednisolone. The first group was operated with conventional CPB, while the second group was operated with minimally invasive CPB (mini-CPB). We detected enhanced level of IL-10 during surgery and at the end of surgery in both groups of patients. While no correlation between IL-10 and IL10R was found, IL-10 was positively correlated with increased percentage of neutrophils at the time points when the level of IL-10 peaked.

## 1. Introduction

Cardiac surgery is connected to profound inflammatory response, characterized by increased level of both proinflammatory and anti-inflammatory mediators. Cardiopulmonary bypass (CPB) is considered to be a potential trigger of cytokine release, therefore, the impact of cardiac surgery on morbidity and mortality is often correlated with the use of CPB [1, 2]. Surgery conducted with the use of CPB is accompanied by ischemia-reperfusion injury, and above that, CPB also represents the environment where blood cells become activated by contact with artificial surfaces, direct air-contact, and also nonturbulent flow. The effort to remove the harmful effect of conventional CPB has led to a development of new surgical devices and techniques. Mini-CPB, that has recently been introduced and successfully used, is designed to reduce blood cell activation: it provides decreased area of extracorporeal circuit, reduces priming, and also lessens air-blood interface. Another favorable feature of mini-CPB is a biocompatible coating that induces higher tolerance by blood cells. The impact of mini-CPB on suppressing inflammatory response is already described and published [3, 4].

As the blood reaches all the body compartments, inflammatory response to surgical injury exceeds local reaction and becomes systemic. Although the increased production of cytokines is essential for protection against infection and also healing the wounds, unregulated inflammatory response is likely to have a harmful effect. Regulating mechanisms consist of anti-inflammatory molecules such as cytokines, cell surface enzymes, receptor antagonists, and soluble receptors [5]. The net impact of the produced cytokines, whether with proinflammatory or anti-inflammatory properties, is determined by the ability of the immune system to balance the inflammatory response properly [6].

IL-10 is a regulatory and immunosuppressive cytokine that is produced by a variety of cells [7]. Upon binding to its receptor (IL-10R), IL-10RB subunit transmits a signal into the cell which activates STAT3- and STAT3-resposive genes [8]. As a result, the production of proinflammatory cytokines, such as IL-1 and TNF-*α*, is inhibited [9].

The changes in production of IL-10, IL-1, and TNF-*α* have already been extensively evaluated in patients undergoing CABG surgery with conventional CPB [10]. However, the information about kinetics of IL-10 in mini-CPB patients is sparse. We compared the changes of IL-10 serum level, the expression of IL-10 membrane receptor, and the percentage of neutrophils in conventional and mini-CPB patients, as well as between these groups of patients. We also examined the incidence of sepsis and acute renal dysfunction in both groups.

## 2. Material and Methods

### 2.1. Patients

Forty-four patients, undergoing elective coronary artery bypass grafting (CABG) surgery on an arrested heart using CPB, were enrolled into our study. All patients were well informed about the purpose of the study and they confirmed their unconstrained participation by a written consent. The study project was approved by the Ethics Committee of the University Hospital in Hradec Kralove, Czech Republic. Patients, included in the study group in the period from December 2006 to December 2007, were randomly assigned to surgery either with the use of conventional CPB (*n* = 22) or mini-CPB (*n* = 22). Exclusion criteria consisted of acute inflammation, urgent operation, reoperation, combined operations, operative risk more than 5% (according to logistic Euroscore), preoperative level of serum creatinine above 130 *μ*mol/L, hepatic disease, and malignancies. The demographic and preoperative data of our patients are shown in Table 1.

### 2.2. Conventional Cardiopulmonary Bypass (CPB)

CPB was established using a two-stage venous drainage and ascending aortic return. A roller pump (Stöckert Instrumente GmBH, München, Germany), a membrane oxygenator (Dideco SrL, Mirandola, Italy) in a closed modification with a collapsible reservoir, a cardiotomy suction device, and a 40 *μ*m arterial line filter (Dideco SrL, Mirandola, Italy) were integrated into the extracorporeal circuit. The system surface was not treated with any hemocompatible substance. The priming solution consisted of 500 mL of Ringer's lactate, 500 mL of Rheodextran (Rheomacrodex), 5,000 IU heparin, 80 mL of Natrium Bicarbonate (NaHCO_3_ 8,4%), 20 mL of 10% Magnesium Sulphate, and Mannitol (at 1 g/kg body weight). The priming volume was calculated so that hematocrit level reached above 0.22. Heparin was administered intravenously at 300 IU/kg body weight to maintain an activated clotting time (ACT) above 480 s during bypass procedure. Patients received neither aprotinin nor corticosteroids intravenously. Pump flow rates averaged 2.4 L/min/m^2^ of body surface area with pressure maintained at 50–60 mmHg. The patients were kept normothermic. Cardioplegic arrest was induced with a cold blood cardioplegic solution, which consisted of blood mixed with St. Thomas solution (Ardeapharma, Sevetin, Czech Republic) in 4 : 1 ratio. It was administered antegradely into the aortic root with doses added every 20 min or as needed. All patients received an internal artery mammary graft to the left anterior descending coronary artery. The central aortovenous anastomoses were performed during the reperfusion phase of cardiopulmonary bypass with the heart beating. After the termination of CPB, heparin anticoagulation was antagonized using Protamine Sulphate at a dose of 1 : 1.

### 2.3. Minimally Invasive Cardiopulmonary Bypass (Mini-CPB)

Mini-CPB was established using a small 22F two-stage venous drainage and ascending aortic return. Minisystem Synergy (Sorin Group SrL, Mirandola, Italy) consisted of a centrifugal pump, membrane oxygenator, 40 *μ*m arterial line filter, and a venous bubble trap. Cardiotomy suction was not used. The whole system, consisting of a closed loop with the surface treated with PH.I.S.I.O phosphorylcholine coating (Sorin Group SrL, Mirandola, Italy) and very short tubing, was placed close to the operating field. The priming solution, heparinization, pump flow, temperature, and surgery technique were identical with the conventional CPB procedure described above. Cardioplegic arrest, induced according to the Calafiore warm blood-cardioplegia protocol, was administered antegradely into the aortic root. At the beginning of CPB, crystalloid priming solution was flushed retrogradely together with the blood coming from the arterial line to minimize the hemodilution of the patient.

### 2.4. Anesthesiological Management

All patients were anesthetized according to the current protocol of our department. Anesthesia was induced using Thiopental and Midazolam. Muscular relaxation was achieved with Cisatracurium. Anesthesia was maintained with Isoflurane and intermittent use of Sufentanyl. Continuous infusion of Propofol was used as a supplement if needed. Volume-controlled ventilation with FiO_2_ 0.5 was employed. Mean arterial pressure was kept above 50 mmHg, with norepinephrine administered as required.

### 2.5. Sample Collection and Data Acquisition

Blood samples were withdrawn from subclavian vein before and during surgery and from antebrachial vein in postsurgery period. The samples were collected into anticoagulant-untreated Vacutainer tubes as well as heparinized Vacutainer tubes (Becton Dickinson, UK) at the following time points: before surgery (introduction of anesthesia), at the beginning of CPB, at termination of CPB, at the end of surgery, and on the 1st, 3rd, and 7th postoperative day.

Serum was separated from the blood cells by centrifugation at 1000 g for 15 min. Samples were stored at −80°C prior to analysis. IL-10 concentration was determined by enzyme-linked immunosorbent assay (ELISA), with a sensitivity of 1 pg/mL. ELISA kit was purchased from Bender MedSystems (Austria). IL-10 concentrations were measured using spectrophotometer (Labsystems Multiskan RC, USA) with Genesis software.

Heparinized blood samples were processed immediately after collection. 50 *μ*L of blood was incubated with titrated monoclonal antibodies anti-CD3 FITC/IL-10R PE/CD14 PerCP/CD16 APC. Antibodies, except for anti-IL-10R, were purchased from BD Biosciences (USA), while anti-IL-10R was purchased from BioLegend, USA. Following the 20 min incubation, red blood cells were lysed by hypotonic lysis and then the samples were washed. The data acquisition was performed with FACS Calibur flow cytometer using CellQuest software (BD Biosciences, USA), and the analysis was done using FlowJo software (Tree Star, USA). Expression of IL-10R on cells was displayed as median fluorescence intensity (MFI). Expression of IL-10R during surgery was not assessed.

### 2.6. Statistical Analysis

Values of IL-10, IL-10R, and percentage of neutrophils during and after surgery were compared to preoperative values. Normal distribution of the data was determined by Shapiro-Wilk test. Changes within a group were evaluated using ANOVA for repeated measures and Dunnett's test or Friedman ANOVA and Wilcoxon paired test. To determine the differences between both groups of patients, values of IL-10, IL-10R, and percentage of neutrophils were compared at matching time points using two-way ANOVA for repeated measures and Fisher's LSD test or Mann-Whitney *U* test. The relationship between IL-10 and percentage of neutrophils was assessed by Spearman correlation coefficient. Demographic and clinical data were analyzed by Fisher exact test, Mann-Whitney *U* test, and Student's *t*-test. All tests were performed at a significance level of 0.05. Bonferroni correction was used in case of multiple comparisons. Results of statistical analysis were expressed as medians unless stated otherwise.

## 3. Results

### 3.1. Differences in IL-10 in Serum within the Groups

**Figure 1:**
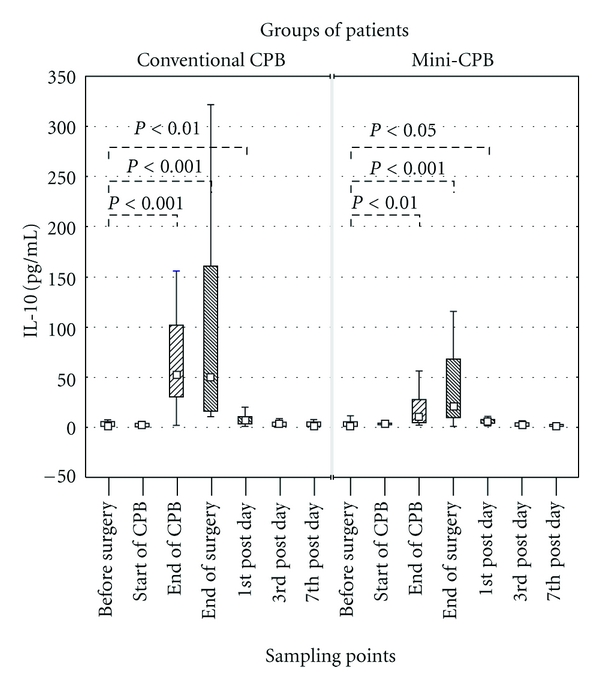
IL-10 level in serum of patients operated either with conventional CPB or mini-CPB. The IL-10 level was detected by ELISA. The level of IL-10 differed between conventional CPB and mini-CPB group after termination of CPB (*P* < 0.01). Even though the difference at the end of surgery was not statistically significant, IL-10 greatly varied when comparing both groups. Squares display median, boxes are quartiles, and whiskers display the range of nonoutlier values.

Changes in IL-10 serum level were similar in both groups of patients, with respect to the preoperative serum level of 1 pg/mL in conventional CPB group and 1.45 pg/mL in mini-CPB group. In the conventional CPB group, serum level of IL-10 significantly increased at the termination of CPB (52.7 pg/mL, *P* < 0.001), at the end of surgery (50.5 pg/mL, *P* < 0.001), and on the 1st postoperative day (7.3 pg/mL, *P* < 0.01). Similarly, the mini-CPB group experienced an increase in serum level at the termination of CPB (from 1.5 pg/mL to 10.6 pg/mL, *P* < 0.01), at the end of surgery (21.2 pg/mL, *P* < 0.001), and on the 1st postoperative day (5.8 pg/mL, *P* < 0.05). Postsurgery monitoring revealed that the level of IL-10 was gradually decreasing in both groups and did not significantly differ from preoperative levels (Figure 1).

### 3.2. Differences in IL-10 in Serum between the Groups

There was significant serum-level difference (*P* < 0.01) between both groups at the termination of CPB: the conventional CPB group topped the mini-CPB group by reaching the value of 52.7 pg/mL. At this time point, IL-10 in mini-CPB group was enhanced, but only reached 10.6 pg/mL. In mini-CPB group, the level of IL-10 was highest at the end of surgery (21.2 pg/mL). In postsurgery period, the level of IL-10 decreased and did not differ between both groups (Figure 1).

### 3.3. Percentage of Neutrophils

**Figure 2:**
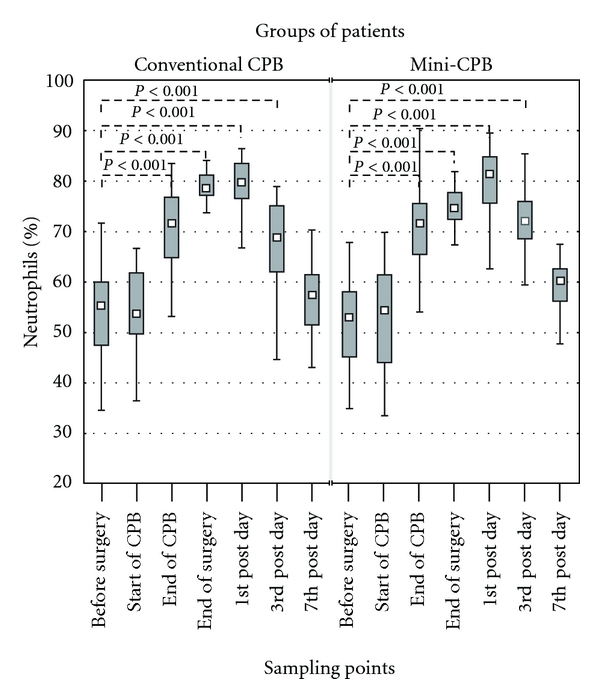
Percentage of neutrophils in peripheral blood samples of patients with conventional CPB or mini-CPB. The percentage of neutrophils was evaluated by flow cytometry. The percentage of neutrophils significantly differed between both groups of patients at the end of surgery (79% of neutrophils in conventional CPB group versus 75% of neutrophils in mini-CPB group, *P* < 0.05). Squares display, median, boxes are quartiles, and whiskers display the range of nonoutlier values.

Percentage of neutrophils was significantly increased (*P* < 0.001) in both groups of patients at the end of CPB, at the end of surgery, and also on the 1st and 3rd postoperative day. The baseline was 55% of neutrophils in conventional CPB group and 53% in mini-CPB group. The highest percentage of neutrophils was measured on the 1st postoperative day in both groups of patients (80% in conventional CPB group and 81% in mini-CPB group). There was statistically significant difference in percentage of neutrophils between both groups at the end of surgery (*P* < 0.05) when conventional CPB group reached 79% and mini-CPB group reached 75% of neutrophils (Figure 2).

**Figure 3:**
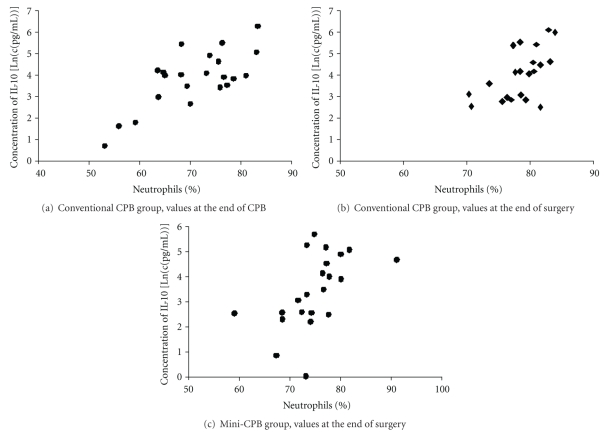
Relationship between percentage of neutrophils and serum level of IL-10. (a) Correlation, *r*_*s*_ = 0.73, in conventional CPB group at the termination of CPB (*P* < 0.001). (b) Correlation, *r*_*s*_ = 0.54, in conventional CPB group at the end of surgery (*P* < 0.01). (c) Correlation, *r*_*s*_ = 0.54, in mini-CPB group at the end of surgery (*P* < 0.01)

The percentage of neutrophils correlated with the maximum level of IL-10 at the termination of CPB (*r*_*s*_ = 0.73, *P* < 0.001), at the end of surgery in conventional CPB group (*r*_*s*_ = 0.54, *P* < 0.01), and at the end of surgery in mini-CPB group (*r*_*s*_ = 0.54, *P* < 0.01) (Figures 3(a)–3(c)).

### 3.4. Expression of IL-10 Receptor (IL-10R)

Since IL-10R is expressed on T lymphocytes, neutrophils, and monocytes, the expression was statistically evaluated in all of these populations. In lymphocytes and neutrophils, no differences were found at any time point when comparing MFI values within a group of patients or between both groups of patients.

**Figure 4:**
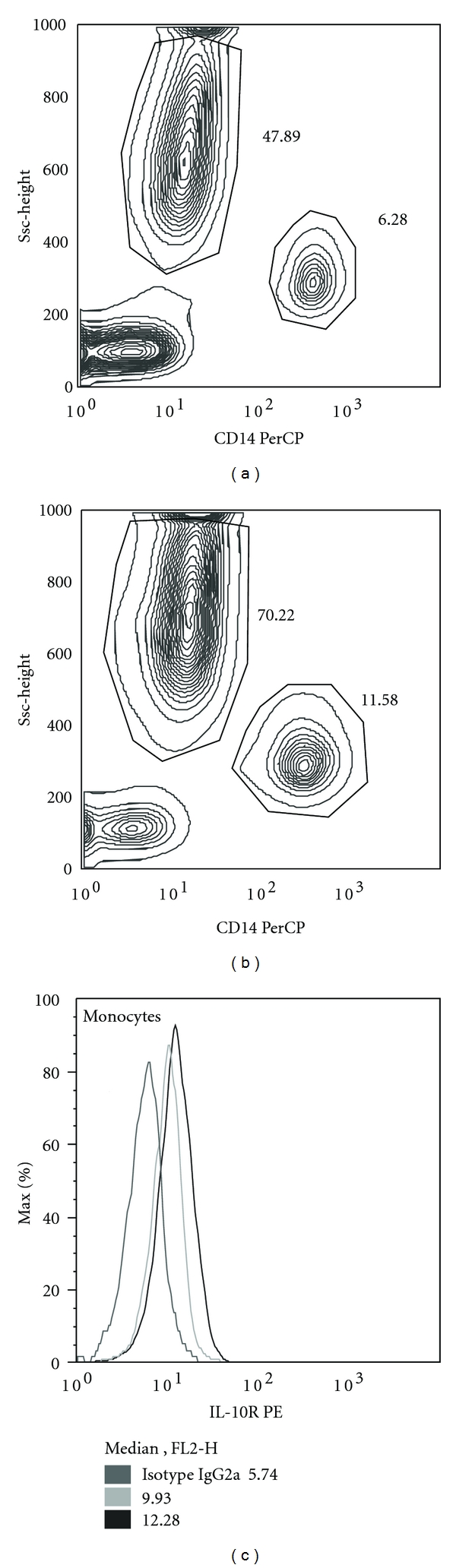
Illustration of changes in the expression of IL-10R on monocytes of conventional CPB patient. Monocytes are discriminated from other populations of cells by high expression of CD14 (*x*-axis). (a) Leukocytes before surgery. (b) Leukocytes on the 3rd postoperative day. (c) Overlaid histograms of monocytes from (a) and (b). Although the expression of IL-10R on monocytes enhances on the 3rd day after surgery (black line), it is relatively weak considering the isotype control (dark grey line). Intracellular staining did not reveal higher expression of IL-10R in cells (data is not shown).

**Figure 5:**
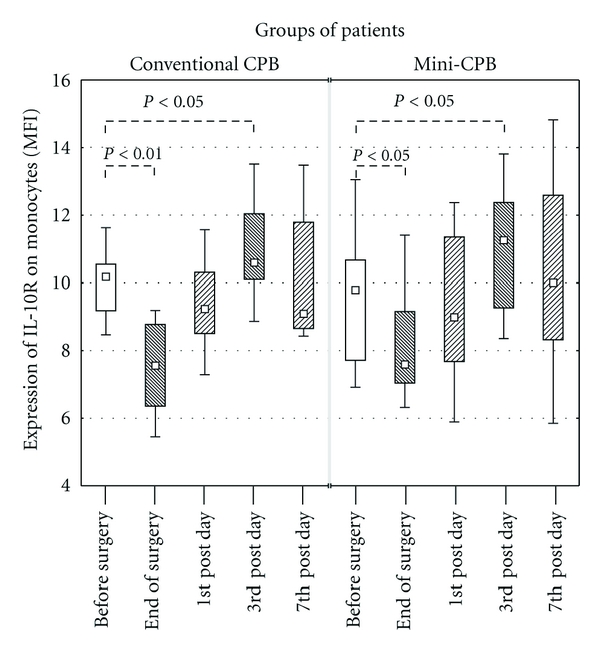
Expression of IL-10R on monocytes in peripheral blood samples of conventional CPB or mini-CPB group as a result of flow cytometry data analysis. The expression of IL-10R did not significantly differ between both groups of patients. Squares display median, boxes are quartiles, and whiskers display the range of nonoutlier values.

The expression of IL-10R on monocytes significantly decreased at the end of surgery in both groups: the preoperative expression of IL-10R dropped from MFI of 10.2 to 7.6 (*P* < 0.01) in conventional CPB group, as well as in mini-CPB, where the preoperative level dropped from MFI of 9.8 to 7.6 (*P* < 0.05). On the 3rd postoperative day, the expression of IL-10R on monocytes was significantly enhanced (Figure 4), reaching MFI of 10.6 in conventional CPB (*P* < 0.05) and 11.3 in mini-CPB (*P* < 0.05). After that, the IL-10R expression decreased again and there was no significant difference between the preoperative value and the value on the 7th day after surgery in both groups (Figure 5).

There was no correlation between serum level of IL-10 and expression of IL-10R on monocytes.

No difference in IL-10R expression on monocytes was observed when comparing both groups of patients (Figure 5).

We observed single- as well as multiple-organ dysfunction or failure in both groups of patients. IL-10 level in each group is listed in Table 2. Only two patients were considered suffering from sepsis. Intraoperative and postoperative data of patients are displayed in Table 3.

## 4. Discussion

IL-10 is an important cytokine, maintaining the proinflammatory response balanced. Although IL-10 can keep cells unresponsive, receptors on the cells affected by IL-10 are still capable of binding and internalizing proinflammatory cytokines. As a result, the cytokines are removed from blood circulation without subsequent activation of the cells [11, 12]. This kind of decoy activity is thought to be of favorable contribution of IL-10 in ischemic heart after myocardial reperfusion, and it is likely to prevent against heart failure [13]. However, high level of IL-10 can also have an adverse effect by increasing the likelihood of sepsis. Nonetheless, a significant association between sepsis and higher level of IL-10 determined by—1082 base pair single polymorphism in promoter region of IL-10 gene is equivocal [14, 15]. It has been reported that CPB induces the release of IL-10, which can be further enhanced by the use of corticosteroids in patients undergoing CABG [16]. In our study, corticosteroids were not applied, and yet we observed that CPB induced a profound release of IL-10. We found significant difference in IL-10 production between the two different types of CPB used. Mini-CPB, which is considered to be a less harmful approach of cardiac surgery, elicited a lower release of IL-10. In spite of this fact, the increase of IL-10 does not seem to impact clinical outcome. Although the level of IL-10 was lower in mini-CPB group, the number of patients suffering from organ dysfunction or failure was nonsignificantly higher in mini-CPB group than in conventional CPB group (*P* < 0.22) (Table 2). While we would be able to predict organ dysfunction or failure according to IL-10 level in conventional CPB group, the similar relation was unclear in mini-CPB group. We also looked at the IL-10 value in patients with sepsis and patients without sepsis. Microbiologically confirmed, there were only two septic patients, one in each group, both reaching values of IL-10 in serum that fell within quartiles of IL-10 calculated for each sampling time point in any given group. Therefore, in our study groups, even though the IL-10 level was very high in some patients, IL-10 could not be used as a predictive marker related to sepsis. Increased level of IL-10 along with the medical treatment (Ampicillin-sulbactam) of our patients might have prevented the onset of sepsis. Such a beneficial role of IL-10 is in concordance with experimental studies [17, 18].

In previous works, IL-10 level was also found to be significantly increased in patients who suffered from postoperative renal dysfunction [19], which was characterized by creatinine level raised above 176 *μ*mol/L [20]. The mini-CPB group had two patients that were suffering from acute renal dysfunction, while there was only one patient in conventional CPB group. The patient from conventional CPB group reached 522.5 pg/mL at the termination of CPB. This patient had the highest value of IL-10 in serum out of both groups. However, the other two patients did not exceed quartiles of IL-10 calculated for each sampling time point. According to this result, there is no simple relation between enhanced level of IL-10 and increased probability of acute renal dysfunction.

We compared the percentage of neutrophils to IL-10 level when IL-10 in serum was at its highest level. We observed significant correlation between IL-10 and percentage of neutrophils at the termination of CPB in conventional CPB group (*r*_*s*_ = 0.73, *P* < 0.001) and at the end of surgery in mini-CPB group (*r*_*s*_ = 0.54, *P* < 0.01). We also found lower—but still significant—correlation (*r*_*s*_ = 0.54, *P* < 0.01) between IL-10 and percentage of neutrophils at the second highest level of IL-10 (at the end of surgery) in conventional CPB group. Our data suggests that neutrophils were the main producers of IL-10 in most of our cardiac surgical patients. However, the lower correlation coefficient at the end of surgery indicates other cells might also have participated in IL-10 production. The observation is in agreement with recently published findings that show that neutrophils are an important source of IL-10 [21] and that mini-CPB attenuates neutrophil activation and cytokine release after coronary bypass surgery [3].

IL-10 exerts its function through the binding to IL-10R [22]. It seems that higher level of IL-10 in conventional CPB group is exclusively linked to the surgery technique and devices used. Since we found no significant difference in expression of IL-10R between both groups of patients, we can hypothesize that the expression of IL-10R on hematopoietic cells exceeds the maximum level of its ligand, IL-10. It has been discovered that other cells, such as fibroblasts and epithelial cells [23, 24], also express IL-10RA after induction; thus IL-10 may have a much broader effect on tissues and organs, an effect which cannot be explained by the possible correlation of serum IL-10 and the expression of IL-10R on monocytes.

## 5. Conclusion

IL-10 level and percentage of neutrophils are significantly affected by the type of cardiac surgery employed. Although IL-10 level may have statistical relation to sepsis or renal dysfunction, the generally accepted critical level that would enable to unambiguously distinguish between patients with worse or good prognosis does not exist. However, in certain conditions, like using conventional CPB, IL-10 may represent a supporting tool, that, along with other parameters, would help rank the patients in likelihood of organ dysfunction or failure. The observed correlation between increased level of IL-10 and higher percentage of neutrophils in both groups of patients suggests functional relationship between both parameters.

## Figures and Tables

**Table 1:** Demographic and preoperative data.

	Conventional CPB		Mini-CPB		*P* value
Patients (no.)	22		22		
Women/men (no.)	4/18		2/20		0.66
Age (years)	68	(63–71)	69	(66–74)	0.57
Body mass index	28.5	(25.3–32)	26.5	(25–31.3)	0.38
Acetylsalicylic acid (no.)	21		21		
Beta blockers (no.)	21		20		
ACE inhibitors (no.)	16		18		0.72
Statins (no.)	21		20		
Diabetes mellitus (no.)	4		6		0.72
COPD (no.)	3		4		
Prior myocardial infarction (no.)	12		9		0.37
Leukocytes (cells ×10^9^/L)	7.5	(6.8–8.5)	7.1	(6.3–8.8)	0.8
Ejection fraction (%)	64.5	(51.3–69.8)	60	(48.5–69.5)	0.35

Parameters marked as “no." display the number of positive cases in a group of patients or number of patients treated with a given medication. Other parameters are characterized by median value and interquartile range in brackets. If both groups contain the same number of cases or if they unequal by a case, then *P*-value is above 0.99 and is not displayed. ACE: angiotensin-converting enzyme; COPD: chronic obstructive pulmonary disease.

**Table 2:** IL-10 level in groups of patients without and with organ dysfunction or failure.

	No organ affected (*n* = 15)	One and more organs affected (*n* = 7)	One organ affected (*n* = 5)	More organs affected (*n* = 2)
Conventional CPB (*n* = 22)	89 (41−451)	203 (148−731)	181 (148–385)	467 (203−731)

	No organ affected (*n* = 11)	One and more organs affected (*n* = 11)	One organ affected (*n* = 6)	More organs affected (*n* = 5)

MINI-CPB (*n* = 22)	35 (13−219)	75 (23−192)	105 (23–183)	55 (49–192)

Groups of patients were divided by number of organs suffering from dysfunction or failure. Upper value displays median of IL-10 (pg/mL), values in the brackets describe the range from minimum to maximum characterizing a group.

**Table 3:** Intraoperative and postoperative data of patients.

	Conventional CPB		Mini-CPB		*P* value
Duration of surgery (min)	210	(161–250)	165	(155–203)	0.15
Duration of CPB (min)	70	(56–111)	62	(55–76)	>0.1
Priming solution (mL)	1600	(1425–1800)	1100	(1000–1300)	<0.001
Anastomoses (no.)	2	(2-3)	2	(2-3)	0.7
Intraoperative blood loss (mL)	1000	(725–1000)	700	(500–950)	0.14
Postoperative blood loss in 24 h (mL)	650	(450–1075)	600	(500–950)	>0.1
Acute renal dysfunction and failure (no.)	1		2		
Respiratory dysfunction and failure (no.)	4		9		0.19
Postsurgery myocardial dysfunction and AMI (no.)	5		6		
Sepsis (no.)	1		1		

Parameters marked as “no." display the number of positive cases in a group of patients with the exception for number of anastomoses which denotes median value. All other parameters are characterized by median value and interquartile range in brackets. If both groups contain the same number of cases or if they unequal by a case, then *P*-value is above 0.99 and is not displayed. AMI: acute myocardial infarction.
